# Effect of technology-aided training on physiological and psychological sports performance: Moderation analysis of sport involvement

**DOI:** 10.1371/journal.pone.0325885

**Published:** 2025-06-24

**Authors:** Yung-Chang Yu, Chen-Yueh Chen, Wen-Cheng Chen, Yen-Kuang Lin, Shu-Cheng Lu

**Affiliations:** 1 Department of Physical Education, Fu Jen Catholic University, New Taipei City, Taiwan; 2 Doctoral Program of Transnational Sport Management and Innovation, National Taiwan Sport University, Taoyuan City, Taiwan; 3 Graduate Institute of Athletics and Coaching Science, National Taiwan Sport University, Taoyuan City, Taiwan; 4 Taoyuan City Pingzhen Civil Sport Center, Taoyuan City, Taiwan; King Faisal University, SAUDI ARABIA

## Abstract

This study investigates the impact of technology-assisted sports training on the physiological and psychological performance of recreational exercisers (non-athletes), with particular attention to the moderating role of sport involvement (SI). A quasi-experimental design was employed, with 48 participants randomly assigned to either an experimental group (technology-assisted training) or a control group (traditional coaching) for an eight-week training program. Performance measures included exercise self-efficacy (ESE) and squat speed (SS). Data were analyzed using ANCOVA and linear mixed models. The results showed that technology-assisted training significantly improved SS (p = 0.026), but had no significant effect on ESE (p = 0.905). Furthermore, SI moderated the relationship between training method and ESE: participants with low SI demonstrated significant improvements in ESE under traditional coaching (p = 0.006), whereas those with high SI showed no significant differences between training methods. These findings suggest that while sports technology can enhance physical performance, it does not necessarily improve exercise self-efficacy. For individuals with low sport involvement, traditional coaching remains essential, highlighting the importance of combining technology with interpersonal interaction. Future training strategies should be customized according to participants’ levels of sport involvement to optimize both performance and psychological motivation, thereby promoting broader health engagement and exercise participation.

## Introduction

With the rapid advancement of digital technologies, sports technology has become an integral part of modern athletic training. Its applications range from wearable devices to virtual reality (VR), artificial intelligence (AI), and machine learning (ML) [1,2]. These technologies enable precise data tracking and real-time feedback, aiding professional athletes and coaches in optimizing training programs and minimizing the risk of injury [3,4]. However, most existing research on sports technology centers on professional athletes, coaches, and sports scientists, with research indicators primarily focusing on physiological metrics (e.g., heart rate, blood oxygen levels, muscle strength) and performance outcomes (e.g., competition results) [5–9]. Studies involving recreational exercisers have largely concentrated on participation rates or health monitoring applications [1,3,10], with limited exploration of their performance outcomes or exercise self-efficacy (ESE). This study focuses on recreational exercisers, examining the effects of technology-assisted training on both performance and ESE. It aims to fill a critical gap in the literature by providing empirical evidence on the role of sports technology in everyday training for non-athletes, thus supporting broader efforts in health promotion among this population.

Given that sports technology can enhance training effectiveness for recreational users leading to increased satisfaction and improved exercise experiences [9], it is essential to investigate whether such technology can also improve ESE, thereby encouraging sustained physical activity. ESE refers to an individual’s confidence in their ability to engage in and overcome challenges in exercise, which plays a crucial role in initiating, maintaining, and managing a regular exercise routine [11]. For recreational exercisers, real-time feedback and self-monitoring enabled by sports technology not only improve awareness of personal performance [5,9,12] but may also boost confidence in facing physical challenges [2,13,14]. Nonetheless, research on how sports technology may effectively enhance ESE in recreational exercisers remains limited. Thus, a deeper investigation is needed to expand its application among the general population.

As sports technology becomes increasingly widespread, its effective use among recreational exercisers poses new challenges. Unlike professional athletes—who are generally more adaptable—recreational users often encounter difficulties due to the complexity of devices or usability barriers [14]. If the technology is not tailored to users’ varying needs and preferences, it may hinder its effectiveness and reduce users’ confidence and motivation to continue exercising. Therefore, designing and applying sports technologies based on users’ levels of sport involvement (SI) should be considered a key strategy in promoting their adoption.

The significance of this study lies in examining the specific effects of sports technology on enhancing ESE in recreational exercisers while also analyzing the moderating role of SI. Previous research has demonstrated that SI influences exercise behavior and willingness to participate [15]. While SI has been associated with psychological outcomes such as well-being and loyalty [16], few studies have investigated whether technology-assisted training affects exercise performance and ESE across varying levels of SI.

SI is a multidimensional construct comprising several components that influence an individual’s engagement in physical activity [15,17,18]. Prior studies have identified three primary dimensions of SI: attraction, centrality, and social bonding [15]. Attraction pertains to intrinsic motivation and enjoyment, which may influence the willingness to incorporate technology into training routines [15]. Centrality reflects how deeply exercise is embedded in one’s lifestyle and identity, shaping long-term behavioral patterns [17,18]. Social bonding relates to interpersonal relationships and community connections that sustain engagement and encourage technology adoption [15]. Understanding these dimensions is essential for evaluating how SI moderates the effectiveness of technology-assisted training. For example, individuals with high attraction may be more receptive to using sports technology, while those with strong social bonds may engage more if the technology facilitates group interaction (e.g., virtual workouts). Despite these theoretical insights, little research has empirically examined how these SI dimensions interact with technology use and ESE.

Although some empirical studies suggest that SI moderates psychological variables in different sports contexts [17,18], its role in influencing the relationship between technology-assisted training, exercise performance, and ESE has not been thoroughly explored. Whether previous findings in related areas are applicable in this context remains unclear. Therefore, investigating SI as a moderator warrants further research to broaden the scope of sports technology applications.

To address these gaps, the present study aims to assess both psychological and performance-related outcomes of sports technology use, focusing specifically on ESE and SS. While prior studies have largely emphasized the physiological benefits of sports technology, relatively few have explored its impact on motivation and exercise adherence. At the same time, although SI has been widely acknowledged as a predictor of exercise participation, its moderating influence in technology-assisted contexts remains understudied. This research seeks to bridge these gaps by: (1) evaluating whether sports technology improves ESE and SS performance, and (2) investigating whether SI moderates these effects across varying levels of user engagement. By addressing these questions, this study contributes to a more nuanced understanding of the interaction between technology and individual psychological characteristics in optimizing training outcomes and long-term commitment to exercise. Moreover, the findings will inform the development of more tailored and user-friendly sports technologies, ultimately promoting sustained engagement and improved health outcomes among recreational exercisers.

## Literature review

### Exercise self-efficacy

Exercise self-efficacy (ESE) refers to an individual’s belief in their ability to perform a specific behavior in order to achieve a desired goal, and it plays a central role in behavior change theories [19]. According to Bandura’s Social Cognitive Theory (SCT), self-efficacy is a key determinant of motivation and behavior, shaping the level of effort, persistence, and resilience individuals demonstrate when confronting challenges [20,21]. Within the context of physical activity, ESE specifically reflects an individual’s confidence in their ability to maintain regular exercise in the face of barriers such as fatigue, time constraints, social pressure, or health-related issues [22].

A synthesis of existing research suggests that individuals with high levels of ESE are more likely to initiate and maintain consistent physical activity, making it a critical target in exercise-based interventions [23–25]. These individuals typically exhibit stronger goal-setting capabilities, more effective coping strategies, and greater confidence in overcoming exercise-related barriers. In contrast, low ESE is frequently associated with sedentary behavior, obesity, cardiovascular disease, and other adverse health outcomes [23,25]. Several well-established strategies have been shown to enhance ESE. These include: mastery experiences, where successfully accomplishing incremental training goals helps build confidence over time [11]; vicarious experiences, where observing others successfully engage in exercise activities enhances one’s own belief in their ability [20,26]; verbal persuasion, through encouragement and constructive feedback from coaches or AI-driven digital assistants [9]; and self-regulation, involving self-monitoring, real-time feedback, and personalized goal-setting—components often facilitated by modern sports technologies [12].

In addition, environmental factors such as access to exercise facilities, the presence of social support, and the integration of motivational technologies can significantly influence ESE levels [5,9]. These elements align with SCT’s principle of triadic reciprocal determinism, which posits that personal, behavioral, and environmental factors continuously interact to shape engagement in physical activity [20,21].

### Sports technology training

With the advancement of digital technology, sports technology has become increasingly integrated into athletic training, offering real-time performance monitoring, biomechanical feedback, and AI-driven coaching systems [1,2]. Technologies such as wearable devices, virtual reality (VR), and machine learning (ML) applications enable precise data tracking, thereby improving training efficiency and reducing the risk of sports-related injuries [3,4]. These tools support a more structured and data-informed training approach, yielding significant improvements in physiological indicators such as muscle adaptation, endurance, and explosive power [6,8].

In high-intensity and competitive training contexts, sports technology allows for real-time monitoring of variables including heart rate, blood oxygen saturation, movement kinematics, and recovery status, offering individualized performance analytics [6,7]. For example, velocity-based training (VBT) using devices such as the GymAware linear position transducer enables dynamic adjustment of training intensity in real time, optimizing strength development and mitigating the risk of overtraining [27,28].

Beyond physiological outcomes, sports technology also plays a critical role in enhancing psychological motivation and user engagement. Digital training platforms that incorporate gamification, virtual competitions, and real-time performance comparisons have been shown to increase intrinsic motivation and exercise adherence [29,30], foster social bonding through interactive, team-based workouts [10,12], and strengthen self-efficacy by providing immediate feedback on performance progress and achievements [9]. Based on these insights, the following hypotheses are proposed:

H1a: The use of sports technology devices will enhance participants’ squat speed performance.
H1b: The use of sports technology devices will enhance participants’ exercise self-efficacy.

### Sport involvement

Sport involvement (SI) is a multidimensional construct that captures an individual’s psychological engagement with and perceived importance of physical activity. It influences exercise adherence, behavioral patterns, and receptiveness to sports technology [15,31]. SI is typically conceptualized through three key dimensions: (1) attraction, referring to the intrinsic enjoyment and motivational appeal of physical activity; (2) centrality, which indicates the extent to which exercise is integrated into an individual’s identity and daily life; and (3) social bonding, which encompasses the influence of peer relationships, group participation, and community support in maintaining exercise habits [15].

Research indicates that individuals with high SI demonstrate stronger commitment and loyalty to exercise routines. Those with close ties to fitness communities are more likely to maintain long-term engagement and actively participate in group-based training programs [15,31]. SI also plays a role in determining the impact of sports technology: individuals with high SI often possess intrinsic motivation and self-regulatory strategies, making external technological support comparatively less influential [9,15]. In contrast, individuals with low SI, who may lack internal motivation tend to benefit more from the structure, guidance, and external feedback provided by sports technologies [2,10].

Each SI dimension also moderates the effectiveness of technology-assisted training on ESE. For example, individuals with high attraction toward physical activity are intrinsically motivated and may rely less on technological reinforcement [16]. Those with high centrality are likely to integrate sports technology into their training to reinforce athletic identity and performance [17]. Meanwhile, individuals who score high in social bonding may benefit most from interactive digital features, such as virtual training communities, gamified challenges, and AI-generated feedback, that promote social engagement and real-time self-monitoring, thereby enhancing ESE [9,18].

Considering these interaction patterns, SI is expected to moderate the effects of technology-assisted training on both performance and psychological outcomes. Individuals with different levels of SI are likely to respond differently to technological interventions, leading to variation in ESE and performance development. This is consistent with Bandura’s Social Cognitive Theory (SCT), which posits that self-efficacy, motivation, and prior experiences influence behavioral responses and training outcomes [20,21]. Highly involved individuals may prefer autonomous, self-directed training, whereas those with lower SI may depend more on external feedback and reinforcement [9]. Based on this theoretical foundation, the following hypotheses are proposed:

H2a: Sport involvement moderates the effect of sports technology intervention on squat speed training performance.
H2b: Sport involvement moderates the effect of sports technology intervention on exercise self-efficacy.

## Materials and methods

### Research design and research setting

This study adopts a quasi-experimental design with a pre- and post-test structure to strengthen the validity of causal inferences. The research was conducted at the Pingzhen Civil Sports Center (PZSC), selected as the experimental site due to its extensive engagement in sports technology training. The center’s CEO possesses considerable expertise in the field, and PZSC also serves as a practical site for multiple governmental initiatives, including the Taoyuan Department of Sports’ grassroots athlete programs and the Health Promotion Administration’s recreational training programs. This demonstrates the center’s broad experience in delivering sports technology interventions across various user groups, making it an ideal setting for this study.

**Fig 1:**
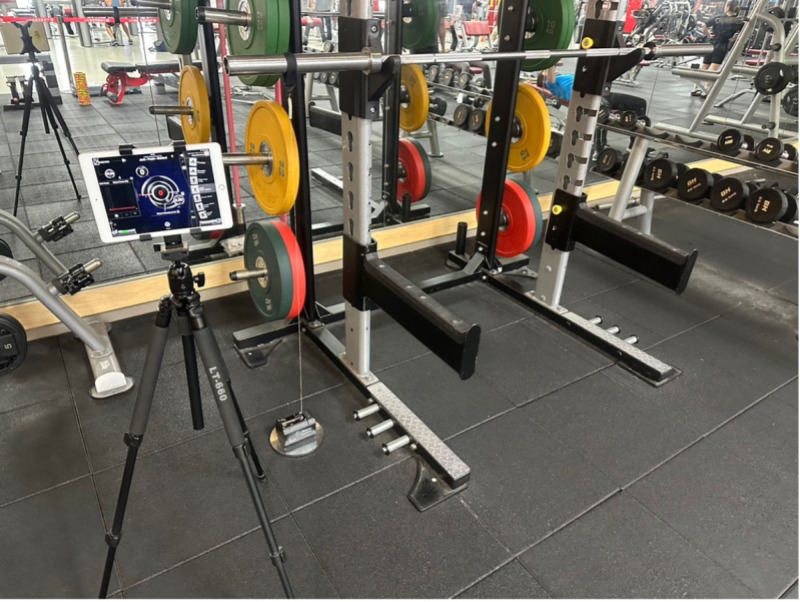
GymAware linear position sensor device.

Participants were assigned to either an experimental group or a control group. The experimental group received training based on Velocity-Based Training (VBT) principles, utilizing GymAware linear position transducers throughout the intervention (Fig 1).

The GymAware optical encoder is a highly reliable and validated Linear Position Transducer (LPT) that accurately measures displacement, mean velocity, peak velocity, and time variables. It also computes key performance indicators such as force and power. Both manufacturer specifications and empirical studies affirm GymAware’s exceptional measurement precision. When tested with a precision calibration device (CalRIG), the system demonstrated a distance error of 0.00 m and a velocity error ranging from 0.01 to 0.02 m/s—indicating excellent accuracy and consistency in capturing displacement and velocity metrics. Additionally, the system has shown strong test-retest reliability (TEM) across different sensors and repeated trials, further validating its measurement integrity.

Compared to traditional training methods that rely predominantly on verbal coaching, this technology-assisted model offers objective, real-time performance data, facilitating more precise and individualized training adjustments. GymAware displays movement velocity instantly and uses built-in algorithms to automatically detect the concentric phase of each exercise repetition. This feature allows coaches or researchers to adjust training intensity in real time based on velocity trends, thereby maximizing training efficacy while minimizing the risk of overexertion. In resistance training contexts, GymAware enables dynamic regulation of load based on velocity feedback, ensuring both the effectiveness and safety of the training protocol.

This study adhered to the American College of Sports Medicine (ACSM) guidelines for foundational strength and conditioning training [32], ensuring that both the experimental and control groups followed identical training schedules and maintained a consistent relative intensity of 70%. This standardization minimized potential confounding effects from environmental variables. However, rather than relying on the traditional one-repetition maximum (1RM) estimation method, this study adopted a dual-verification approach to determine training loads, integrating both RPE and VBT.

The Borg CR-10 RPE scale was used to evaluate subjective training intensity, with sessions prescribed at an RPE level of 7, defined as “very strenuous, but still able to perform a few more repetitions.” This scale is widely used in sports science and clinical settings and has been validated for its strong correlation with physiological markers such as heart rate and blood lactate concentration. A large-scale study by Scherr et al. confirmed the scale’s validity by reporting a high positive correlation between RPE ratings and both heart rate and lactate responses [33].

To further enhance training precision, the experimental group implemented VBT using the GymAware system to monitor mean concentric velocity during squat exercises. According to prior VBT research, a target velocity of 0.82 m/s corresponds to approximately 70% of 1RM in squat training [34]. Participants were instructed to maintain this velocity during repetitions, with real-time velocity data enabling dynamic load adjustments. If a participant’s velocity fell below the target, the resistance was reduced to prevent fatigue accumulation; conversely, if velocity exceeded the target, the load was increased to ensure adequate training stimulus.

In addition to VBT, session-by-session RPE verification was conducted throughout the eight-week training intervention. At the start of each session, participants verbally reported their expected RPE based on prior experiences. After completing the first set of each exercise, they reassessed their perceived exertion. If RPE was below 6, indicating underloading, weight was increased. If RPE exceeded 8, indicating excessive effort, resistance was reduced to mitigate fatigue accumulation.

The experimental group thus received dual-modality training regulation, incorporating both real-time VBT feedback and RPE-based subjective monitoring. In contrast, the control group used RPE ratings alone to regulate intensity and did not receive any real-time velocity feedback. This comparison allowed the study to examine whether integrating VBT would improve training precision, exercise self-efficacy, and SS performance.

By combining subjective (RPE) and objective (velocity) metrics, the study achieved a more comprehensive evaluation of training intensity and fatigue. For instance, when a participant reported a high RPE along with a noticeable decline in movement velocity, it was interpreted as a marker of excessive fatigue. In such cases, coaches could intervene by modifying the load or adjusting rest periods to tailor training to the individual’s needs. This integrative monitoring system not only improved load management but also reduced the risk of overtraining and enhanced overall performance outcomes [33].

**Fig 2:**
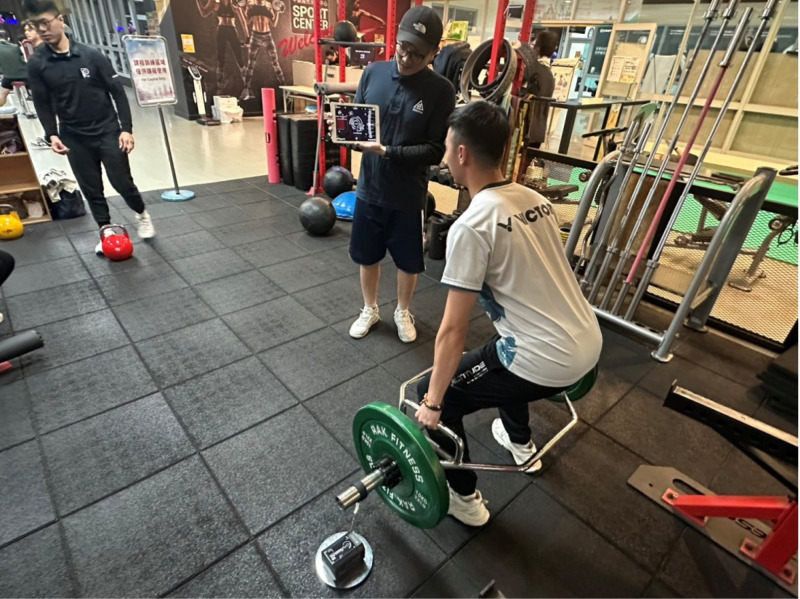
The experimental group uses GymAware training photo illustration.

During training sessions, the GymAware system displayed real-time velocity data on a tablet interface (Fig 2). Coaches monitored the data live and provided immediate recommendations. When average concentric velocity dropped below 0.82 m/s, the load was reduced to prevent overload; when velocity exceeded 0.82 m/s, the load was increased to maintain the targeted intensity. This real-time feedback and expert-guided load adjustment process ensured a balance between scientific measurement and professional coaching judgment, thereby reducing reliance on subjective perception alone [34].

For the control group, training load adjustments were based exclusively on self-reported RPE scores. Participants did not receive access to velocity data or real-time coaching feedback. To compensate for the absence of visual feedback, a standardized verbal instruction “Perform the movement as quickly as possible” was delivered during each session. This directive was intended to elicit maximal voluntary movement velocity, thereby encouraging effort levels comparable to those of the experimental group [33,34]. All training sessions were supervised by certified coaches with academic backgrounds in sports science and a minimum of two years of instructional experience at the PZSC. A standardized coaching protocol was implemented across all sessions to ensure consistency in movement execution, training duration, cueing language, and feedback delivery. This approach minimized participant-level variability while preserving the key experimental distinction between the two groups.

To ensure the internal validity of the intervention, a total of 48 participants were recruited and randomly assigned to either the experimental or control group, maintaining a balanced 1:1 gender ratio within each group. Prior to the intervention, all participants completed a pre-test that included measurements of explosive power (SS) and ESE, as well as the SI scale during the initial session. The post-test was conducted after the final (16th) training session, capturing the impact of the 8-week intervention on both physiological and psychological outcomes. SS was measured in meters per second (m/s) using velocity tracking devices, while ESE and SI scores were assessed using a 5-point Likert scale (1 = strongly disagree; 5 = strongly agree). The 8-week training duration was selected based on both PZSC’s standard course cycle and prior empirical research, which suggests that an 8-week resistance training period is sufficient to elicit measurable changes in muscle adaptation and performance [35,36]. Therefore, the intervention was carried out over eight weeks, with post-testing conducted after the final session to determine whether participants in both groups exhibited significant improvements in physiological (explosive power) and psychological (ESE) indicators. The results of the pre- and post-test correlation analysis, respectively (Table 1).

**Table 1:** Sport involvement and exercise self-efficacy questionnaire analysis.

	Experiment Group (n = 24)	Control Group (n = 24)	Total (n = 48)	p-Value
SI, Mean (SD)	3.75 (0.52)	4.06 (0.65)	3.91 (0.6)	0.072
ESE_T1, Mean (SD)	3.80 (0.29)	3.85 (0.58)	3.82 (0.46)	0.756
ESE_T2, Mean (SD)	3.82 (0.44)	4.12 (0.55)	3.97 (0.52)	0.041^*^

SI, sport involvement; SD, standard deviation; ESE, exercise self-efficacy;

* p < 0.05.

### Research participants and sampling process

Participants in this study were recruited via the official website of the PZSC, its Facebook page, and on-site bulletin boards. To align with the study’s objectives, recruitment was limited to recreational exercisers aged 20–65. Recreational exercisers were defined as individuals who engage in regular physical activity but have not received formal competitive sports training. Individuals under 20 or over 65 were excluded to reduce the influence of age-related variation.

A total of 48 participants were enrolled, with an equal gender distribution: 50% male (n = 24) and 50% female (n = 24). To control for gender-related effects, participants were grouped by gender, and random assignment was conducted within each gender group. This ensured that both the experimental and control groups included 12 males and 12 females each. Regarding age distribution, 40% of participants were aged 40–49, 35% were aged 30–39, and 25% were aged 20–29. No participants over the age of 50 were included in the study.

Before the intervention, all participants completed pre-tests measuring SS (as an indicator of explosive power), ESE, and the SI scale. The post-tests were conducted after the 16th training session to assess the outcomes of the intervention. Although participants were not matched based on external characteristics, random assignment with gender balancing ensured that the baseline characteristics of the experimental and control groups were comparable. The effectiveness of randomization was supported by similar gender ratios and age distributions between the groups (Table 2). In addition, to statistically control for individual differences, pre-test scores for SS and ESE were included as covariates in ANCOVA models, improving the precision of the group comparisons.

**Table 2:** Gender and age distribution of participants in the experiment & control groups.

	Experiment Group (n = 24)	Control Group (n = 24)	Total (n = 48)	p-Value
Gender				
Male (%)	12 (50%)	12 (50%)	24 (50%)	1
Female (%)	12 (50%)	12 (50%)	24 (50%)	
Age				
20 ~ 29 (%)	7 (29%)	5 (21%)	12 (25%)	0.694
30 ~ 39 (%)	9 (38%)	8 (33%)	17 (35%)	
40 ~ 49 (%)	8 (33%)	11 (46%)	19 (40%)	
50 ~ 59 (%)	0 (0%)	0 (0%)	0 (0%)	
60 up (%)	0 (0%)	0 (0%)	0 (0%)	

The questionnaire used in this study comprised three main sections: ESE, SI, and demographic information. Demographic data included gender, age, and other relevant characteristics. Each participant was assigned a unique identification number by the research team. The measurement of ESE was based on the framework proposed by Li et al. [37] and employed the ESE scale originally developed by Kroll et al. [13]. The scales used in this study were first translated into Traditional Chinese by the researchers. To ensure conceptual and linguistic equivalence with the original English version, a back-translation procedure was conducted by bilingual experts. This process confirmed that the translated version retained the original meaning and conceptual integrity. Prior to the formal administration, participants were given time to read through the questionnaire items. The researchers were available to clarify any uncertainties to ensure full comprehension of the items and alignment with the conceptual scope of the original scales.

Construct validity of the ESE scale was evaluated using Principal Component Analysis (PCA), which showed that all 10 items loaded onto a single factor, accounting for 60.70% of the total variance. To assess convergent validity, a correlation analysis was conducted between ESE and the General Self-Efficacy (GSE) scale, yielding a Spearman correlation coefficient of 0.32, indicating a moderate but significant relationship. This suggests that while ESE is related to general self-efficacy, it also captures distinct dimensions specific to exercise contexts. For reliability analysis, the Cronbach’s alpha (α) for the ESE questionnaire was 0.804 at pre-test (ESE_T1) and 0.92 at post-test (ESE_T2), indicating strong internal consistency over time. The Cronbach’s alpha values for each subdimension of the SI scale were also satisfactory, all exceeding the 0.70 threshold: Attraction: α = 0.91; Centrality: α = 0.90; Social Bonding: α = 0.82; Identity Affirmation: α = 0.75; Identity Expression: α = 0.84.

These values demonstrate that the questionnaires used in this study exhibited good reliability across both ESE and SI constructs, respectively (Table 3).

**Table 3:** Items of the questionnaire used in the present study.

Items	M	SD	t
Exercise Self-Efficacy (T1)			
1. That I can overcome barriers and challenges with regard to physical activity and exercise if I try hard enough.	3.90	0.75	35.96
2. That I can find means and ways to be physically active and exercise.	4.10	0.56	51.23
3. That I can accomplish my physical activity and exercise goals that I set.	3.92	0.68	39.97
4. That when I am confronted with a barrier to physical activity or exercise I can find several solutions to overcome this barrier.	3.60	0.74	33.91
5. That I can be physically active or exercise even when I am tired.	3.56	0.77	32.09
6. That I can be physically active or exercise even when I am feeling depressed.	3.73	0.82	31.57
7. That I can be physically active or exercise even without the support of my family or friends.	3.73	0.92	28.19
8. That I can be physically active or exercise without the help of a therapist or trainer.	3.81	0.7	37.51
9. That I can motivate myself to start being physically active or exercising again after I’ve stopped for a while.	3.92	0.77	35.37
10. That I can be physically active or exercise even if I had no access to a gym, exercise, training, or rehabilitation facility.	3.98	0.81	33.96
Exercise Self-Efficacy (T2)			
1. That I can overcome barriers and challenges with regard to physical activity and exercise if I try hard enough.	4.06	0.56	50.14
2. That I can find means and ways to be physically active and exercise.	4.10	0.59	48.02
3. That I can accomplish my physical activity and exercise goals that I set.	4.00	0.62	44.78
4. That when I am confronted with a barrier to physical activity or exercise I can find several solutions to overcome this barrier.	3.94	0.63	43.12
5. That I can be physically active or exercise even when I am tired.	3.77	0.78	33.56
6. That I can be physically active or exercise even when I am feeling depressed.	3.77	0.78	33.56
7. That I can be physically active or exercise even without the support of my family or friends.	3.98	0.79	35.11
8. That I can be physically active or exercise without the help of a therapist or trainer.	4.00	0.83	33.59
9. That I can motivate myself to start being physically active or exercising again after I’ve stopped for a while.	4.06	0.63	44.49
10. That I can be physically active or exercise even if I had no access to a gym, exercise, training, or rehabilitation facility.	4.00	0.65	42.48
Sport involvement			
*Attraction*			
1. Sport is one of the most enjoyable things I do.	4.19	0.76	38.06
2. Sport is very important to me.	4.27	0.71	41.86
3. Sport is one of the most satisfying things I do.	4.15	0.87	32.83
*Centrality*			
4. A considerable portion of my life revolves around sports.	3.81	1.08	24.35
5. Sports play a central role in my life.	3.75	0.96	27.16
6. Changing my preference from sports to any other recreational activity would require substantial rethinking.	3.58	1.03	24.15
*Social bonding*			
7. I enjoy discussing sports with my friends.	4.02	0.86	32.29
8. Most of my friends are in some way connected with sports.	3.69	1.13	22.55
9. Participating in sports provides me with an opportunity to spend time with friends.	3.88	1.06	25.22
*Identity affirmation*			
10. When I participate in sports, I can be myself.	4.21	0.65	44.79
11. I resonate with people and media associated with sports.	3.98	0.76	36.39
12. When playing sports, I do not have to be concerned about my appearance.	3.85	0.87	30.52
*Identity expression*			
13. You can tell a lot about people by seeing them play sports.	3.88	0.67	39.93
14. Sport participation says a lot about who I am.	3.73	0.71	36.55
15. When I participate in sports, others see me the way I want them to see me.	3.63	0.76	32.98

### Research procedures

This study was reviewed and approved by the Institutional Review Board (IRB) of National Taiwan Sport University on August 26, 2024 (Approval No. NTSUIRB-113-050). Prior to the start of the experiment, the researchers fully explained the study’s purpose and procedures to all participants. The study commenced only after participants voluntarily agreed to participate and signed the informed consent form.

Before the training program began, participants completed pre-tests assessing SS and ESE. Two coaches were then randomly assigned to conduct training for the experimental group and the control group, respectively, and remained with their assigned group for the entire 8-week intervention period. Both coaches had more than two years of instructional experience at the PZSC, ensuring familiarity with standardized training protocols and diverse participant characteristics. This consistency in coach qualifications suggests that potential differences in coaching ability can be considered negligible.

Following completion of the training program, post-tests for SS and ESE were administered during the final (16th) session. All experimental data were subsequently compiled by the research team. Participants were informed that they could withdraw from the study at any time without penalty or conditions. The individual whose image appears in Fig 2 provided written informed consent (in accordance with the PLOS consent form) to have their photograph published in connection with this manuscript.

### Statistical procedures

All statistical analyses were conducted using SAS version 9.4 and SPSS version 27. For hypotheses H1a and H1b, we performed ANCOVA, with Group as the between-subjects factor. Pre-test scores of SS and ESE were entered as covariates, and post-test scores were used as the dependent variables. For hypotheses H2a and H2b, linear mixed model (LMM) were used, with Time (pre- vs. post-test) as the within-subjects factor and Group and SI as between-subjects factors. Interaction terms were included to examine the potential moderating effect of SI. The results indicated that only ESE showed a significant three-way interaction (Group × Time × SI).

Prior to conducting parametric analyses, key statistical assumptions were assessed. The Shapiro–Wilk test was used to evaluate the normality of residuals, and Levene’s test was used to assess homogeneity of variances. The threshold for statistical significance was set at α = 0.05. Effect sizes were reported using Cohen’s d for paired-sample t-tests and Cohen’s f for interaction effects in the linear mixed models. These results are presented in Tables 5 and 6 of the revised manuscript.

**Table 5:** Experiment group SS & ESE paired sample t-test analysis.

	t	p-Value	Cohen’s d
Experiment Group SS_T1-SS_T2	−5.61	0.000*	1.06
Experiment Group ESE_T1-ESE_T2	−0.12	0.905	0.02

SS, squat speed (m/s);

* p < 0.05

**Table 6:** Summary of ESE linear mixed model.

Effect	Beta	SE	t	p
time	0.65	0.92	0.70	0.484
Group	1.52	0.86	1.76	0.085
SI_mean	0.44	0.17	2.58	0.013^*^
Group*time	−2.81	1.22	−2.30	0.026^*^
SI_mean*time	−0.18	0.24	−0.73	0.472
SI_mean*Group	−0.33	0.22	−1.51	0.138
SI_mean*Group*time	0.64	0.31	2.05	0.046^*^

SE, standard error;

* p < 0.05. Power = 0.52. Cohen’s f for SI_mean*Group*time = 0.26.

Additionally, the effect size (Cohen’s d) for the Group × Time interaction on ESE was 5.72 (95% CI: 0.690–11.470), and 0.92 (95% CI: –1.200 to 3.090) for SS. For the ANCOVA model, partial η² for the Group factor was 0.03 (95% CI: 0.001–0.170) for SS at post-test, and 0.09 (95% CI: 0.001–0.257) for ESE at post-test and 0.03 (95% CI: 0.001–0.170) for SS post-test.

## Results

This study investigated whether the integration of sports technology devices into exercise training could enhance participants’ SS performance and ESE. Additionally, the study examined whether SI moderated the effects of the intervention on SS and ESE outcomes.

### Results of H1a and H1b testing

To test the hypotheses, a one-way ANCOVA was conducted. The use of sports technology devices during SS training was treated as the between-subjects factor (Group), while pre-test scores (T1) were used as covariates, and post-test scores (T2) of SS and ESE served as the dependent variables. This analysis was used to assess changes after the 8-week, 16-hour intervention. The results indicated that the use of sports technology devices had a significant effect on SS performance (p = 0.026) and a significant effect on ESE (p = 0.045). These findings suggest that the training intervention involving sports technology positively influenced both SS and ESE outcomes (Table 4).

**Table 4:** One-way ANCOVA for group with SS and ESE.

	F	p	Power for Group effect
DV: T2_SS			0.99
T1_SS	135.07	0.000^*^	
Group	5.29	0.026^*^	
DV: T2_ESE			0.53
T1_ESE	0.23	0.634	
Group	4.25	0.045^*^	

DV, dependent variable;

* p < 0.05.

To further validate these results, paired-sample t-tests were conducted. The results revealed that the intervention had a significant effect on SS performance (t = –5.61, p < 0.01), supporting Hypothesis H1a. This demonstrates that the use of sports technology devices significantly improved SS training outcomes. However, the intervention did not have a significant effect on ESE (t = –0.12, p = 0.905), indicating that participants’ self-efficacy was not significantly enhanced using sports technology. Therefore, Hypothesis H1b was not supported (Table 5).

### Results of H2a and H2b Testing

To test this hypothesis, the researchers employed a LMM, with Time (T1 and T2) treated as a within-subjects variable, and Group and SI as between-subjects variables. The analysis revealed a significant three-way interaction effect among SI, Group, and Time for ESE (p = 0.046; Table 6). However, no significant interaction effect was found for SS (p = 0.748; Table 7).

**Table 7:** Summary of SS linear mixed model.

Effect	Beta	SE	T	p
time	−0.11	0.10	−1.08	0.288
Group	−0.04	0.24	−0.17	0.863
SI_mean	−0.02	0.05	−0.42	0.679
Group*time	0.08	0.13	0.64	0.524
SI_mean*time	0.01	0.03	0.32	0.754
SI_mean*Group	0.00	0.06	0.05	0.958
SI_mean*Group*time	−0.01	0.03	−0.32	0.748

SE, standard error;

* p < 0.05. Power = 0.06. Cohen’s f for SI_mean*Group*time = 0.16.

These findings indicate that Hypothesis H2a was not supported, as SI did not moderate the effect of the sports technology intervention on SS training performance.

To further determine whether Hypothesis H2b was supported, the researchers conducted independent-sample t-tests and paired-sample t-tests on ESE scores within the Low and High SI groups. The results revealed that within the control group, ESE significantly improved under the Low SI condition, while no significant differences were observed under any other conditions (Table 8). Furthermore, the post-test (T2) ESE scores in the control group were higher than those in the experimental group under the Low SI condition (Table 9).

**Table 8:** ESE linear mixed model paired sample t-test analysis.

	t	p-Value
Low SI*ExGRP ESE_T1-ESE_T2	0.60	0.561
Low SI*ConGRP ESE_T1-ESE_T2	−3.45	0.006^*^
High SI*ExGRP ESE_T1-ESE_T2	−0.83	0.427
High SI*ConGRP ESE_T1-ESE_T2	0.00	1.000

ExGRP, Experiment Group; ConGRP, Control Group;

* p < 0.05.

**Table 9:** ESE linear mixed model independent sample t-test analysis.

	t	p-Value
Low SI ESE_T1	1.74	0.096
ESE_T2	−2.37	0.027*
High SI ESE_T1	−1.05	0.260
ESE_T2	−0.41	0.684

* p < 0.05

**Fig 3:**
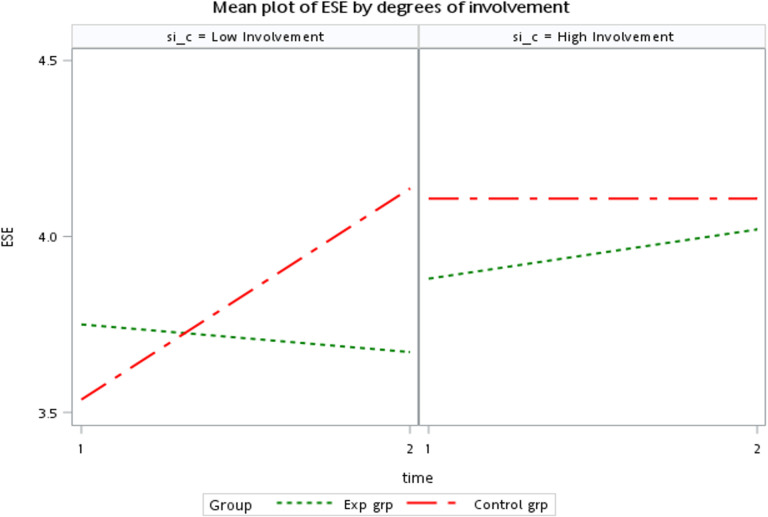
Mean ESE versus the levels of sport involvement.

Taken together, these findings suggest that under the Low SI condition, SI moderated the effect on ESE, specifically among participants in the control group without sports technology intervention. This provides empirical support for Hypothesis H2b (Fig 3).

## Discussion

### General discussion

In this study, the researchers investigated whether the use of sports technology devices in exercise training programs could improve SS performance and ESE, and examined the moderating role of SI in these relationships. The use of an experimental design enabled causal inference, addressing limitations in prior studies that lacked evidence regarding the causal relationship between the use of sports technology devices and ESE. The findings demonstrated that SI moderated the relationship between sports technology use and ESE, revealing variation in ESE outcomes under different usage contexts. Based on these experimental results and related literature, it can be further inferred that SI moderates the psychological effects that emerge under different intervention conditions.

First, the results of H1a showed that the use of sports technology devices effectively enhanced participants’ SS training performance. This is consistent with previous research. For example, wearable technology provides real-time physiological and performance data, allowing coaches to adjust training plans, monitor training loads, and assess whether training intensity is appropriate. This helps improve training precision and individualization [9].

Additionally, the results of H1b indicated that the use of sports technology devices had no significant effect on ESE. While direct empirical evidence connecting these variables remains limited, prior research suggests that the effectiveness of technology in exercise contexts may depend on whether it supports users’ basic psychological needs, such as autonomy and relatedness [10]. When users rely too heavily on sports technology, they may adopt a passive role in their training, allowing the device to dictate goals and routines at the expense of personal agency and self-awareness. As a result, their sense of autonomy and intrinsic motivation may decline, leading to diminished self-efficacy. This explanation is supported by Self-Determination Theory (SDT), which posits that intrinsic motivation is maintained when individuals feel autonomous, competent, and connected to others. If these needs are not met through technology-mediated training programs that are highly structured and externally directed, users may become disengaged from self-regulated exercise behavior [29].

Second, the results confirmed that SI moderated the relationship between ESE and the sports technology intervention. The findings of H2a, however, showed that SI did not moderate the effect of sports technology on SS performance. Although direct evidence on this relationship is limited, related research suggests that individual physiological and psychological traits, such as age, gender, health status, and physical ability, may have a more substantial impact on training outcomes. These factors may reduce the potential for SI to exert a moderating effect [9,38].

Moreover, the results of H2b showed that under the low SI condition, ESE significantly increased in the control group, with post-test scores higher than those of the experimental group. Based on relevant literature, it can be inferred that individuals with low SI tend to lack intrinsic motivation for exercise [12]. Even when using sports technology devices, it may be difficult to stimulate their enthusiasm, making it challenging to enhance ESE effectively. SI is grounded in the concept of personal relevance, which refers to the degree to which an activity is connected to an individual’s self-concept [15]. Individuals with low SI may lack this personal connection. This often manifests as low interest or confidence in exercise, resulting in weaker intrinsic motivation. If sports technology is overly complex, lacks enjoyment, or fails to offer personalized goal setting, it may generate frustration or stress, further undermining ESE.

Additionally, exercise atmosphere and ESE are key factors influencing exercise commitment and adherence. Setting achievable goals can improve self-efficacy and long-term engagement [38]. A positive training environment can provide social support, including coach guidance, peer encouragement, and a sense of group belonging. These forms of support may enhance ESE, especially for individuals with low SI [37]. In this study, the control group did not use sports technology devices. Instead, coaches communicated exercise goals verbally through relative comparisons. For example, participants were encouraged with statements such as, “Let’s try to go faster than before.” Both coaches and participants evaluated goal attainment based on shared subjective perceptions. The researchers inferred that, in the absence of objective data, participants in the control group perceived goal achievement when both they and the coach believed that the effort met the intended target. This may have allowed individuals with low SI in the control group to experience a stronger sense of accomplishment, which in turn enhanced their ESE.

The findings also indicated that participants with high SI did not experience significant improvements in ESE. This may be explained by the ceiling effect. This phenomenon is well documented in self-efficacy research, in which individuals with high baseline efficacy demonstrate limited room for further improvement [19,21]. Previous studies have shown that those with strong baseline self-efficacy may benefit less from interventions, as they have already developed confidence and mastery in their exercise behavior [11,19]. Furthermore, individuals with high SI tend to engage in autonomous goal setting and self-monitoring. This reduces their dependence on external feedback, such as that provided by sports technology [15,16,26,31].

Another possible explanation is the overjustification effect. This occurs when externally imposed feedback reduces the intrinsic motivation of individuals who are already self-driven [9,12,29]. Prior studies have shown that excessive use of external reinforcement, such as real-time tracking, does not always enhance psychological outcomes. This is particularly true for individuals with strong internal motivation. In addition, perceived exertion and training-related fatigue may play a role in limiting self-efficacy improvement. Studies have found that perceived exertion can moderate the psychological impact of training. Individuals with high SI may experience greater fatigue due to higher training intensity, resulting in less perceived psychological gain despite the use of technology [33]. These explanations collectively help clarify the results observed in this study.

### Research and practical implications

This study has important implications for both academic research and practical application.

From an academic perspective, the use of an experimental design provided empirical evidence that sports technology-assisted training can significantly improve physiological performance, such as SS. These results contribute to a better understanding of how sports technology enhances the effectiveness of exercise training, particularly among recreational exercisers. In addition, the study explored the moderating role of SI, revealing that SI influences how individuals respond to sports technology in both physiological outcomes and ESE.

Specifically, individuals with low SI exhibited greater improvements in ESE under traditional coaching, while highly involved individuals showed a weaker psychological response to technological interventions. These findings expand the scope of research on sport psychology and technology use by incorporating psychological engagement as a moderating factor. Finally, this study addresses an existing gap in the literature, which has primarily focused on elite athletes, coaches, or sport science professionals. By centering on recreational exercisers, this research provides valuable empirical data on how sports technology can be applied to the recreational exercisers, thereby enriching the existing body of knowledge.

From a practical standpoint, the findings highlight the importance of personalized design in sports technology. Devices should be adapted to accommodate users with varying levels of SI. For instance, individuals with low SI may benefit from simplified interfaces, interactive features, and achievement-based feedback to enhance motivation. Conversely, highly involved individuals may prefer advanced data analytics and precision training programs to meet their performance-oriented needs. The findings also reaffirm the continued relevance of traditional coaching models. For individuals with low SI, interpersonal interaction, encouragement, and shared goal setting in traditional coaching can significantly enhance ESE. Therefore, rather than replacing human instruction, sports technology should be integrated with traditional guidance to create a balanced and effective training environment.

Finally, the study underscores the potential of sports technology in promoting health and exercise participation among recreational exercisers. The application of such technology should not be confined to elite or competitive settings. When designed with accessibility and usability in mind, sports technology can reduce participation barriers, encourage more people to engage in physical activity, and contribute to broader public health outcomes. In this way, the integration of technology into general fitness practice can play a meaningful role in fostering long-term health behavior change.

### Limitations and directions for future study

This study has several limitations. First, the sample size of 48 participants was relatively small, which limited the feasibility of conducting a confirmatory factor analysis (CFA). Nevertheless, previous studies in the field of sports science have used similarly small sample sizes and have still reported statistically significant results [39–41]. To ensure methodological rigor, an a priori power analysis was conducted based on large effect sizes (Cohen’s d > 0.90) reported in the literature for comparable outcome measures [42]. Using an estimated effect size of d = 0.90, α = 0.05, and a desired power of 0.80, a two-tailed independent-samples t-test indicated that at least 21 participants per group were needed. To account for potential attrition and missing data, the sample size was increased by approximately 10%, resulting in a final sample of 48 participants (24 per group). Additionally, a post-hoc power analysis was conducted to evaluate whether the achieved sample size was adequate. The results indicated that the statistical power for testing hypotheses H1a and H1b was 0.99 and 0.53, respectively, while for H2a and H2b, it was 0.06 and 0.52. The notably low power for H2a (0.06) suggests that the lack of statistical significance may have been due to insufficient sample size rather than the absence of an actual moderating effect. Future studies should consider recruiting larger samples to enable robust confirmatory factor analysis (CFA) and to more comprehensively examine the moderating role of SI in training-related outcomes.

Second, although this study set an age range of 20–65 years for participant recruitment, no individuals aged 50 or older were enrolled. As a result, the final sample, while not explicitly excluding older adults, lacked representation from this demographic. Since age has been shown to influence both ESE and the acceptance of sports technology [11,19], the absence of participants over 50 may limit the generalizability of these findings to older populations. Furthermore, all participants were regular attendees of exercise programs at the PZSC, ensuring prior exposure to structured physical activity. However, individual differences in fitness levels and resistance training experience were not systematically recorded. While this may constrain analyses based on training history, the inclusion of physically active individuals offers consistency in baseline activity levels. Future research should seek a more balanced age distribution to examine whether the observed effects vary across age groups.

Third, although prior studies suggest that an eight-week intervention is sufficient to detect training effects [35,36], future studies should consider extending the duration of the intervention to examine the long-term sustainability of improvements in SS and ESE. A longer timeframe would help determine whether the observed benefits are maintained or diminish once technological feedback is removed. In addition, this study did not employ a blinded design; both participants and coaches were aware of group assignments, which could have introduced expectancy effects. Future research should consider single- or double-blind protocols to minimize potential bias. The absence of a placebo control condition is another limitation. Improvements in ESE may have been influenced, at least in part, by participants’ expectations rather than the actual effects of the technology. Future studies should consider incorporating sham interventions or non-functional control devices to differentiate genuine effects from placebo responses.

Finally, this study utilized only one type of sports technology device and did not account for the potential influence of other technologies such as wearable devices, VR, or AR. These technologies offer varying levels of interactivity, feedback, and motivational features, all of which may impact training adherence and ESE differently. Future research should examine whether the findings of this study can be generalized across different forms of sports technology and investigate how specific types of devices interact with individual differences in SI and motivation.

By addressing these limitations, future research can provide a more nuanced and comprehensive understanding of how sports technology impacts training outcomes across diverse populations, devices, and contexts.

## Conclusions

The results of this study indicate that sports technology-assisted training significantly enhances exercise outcomes, particularly through improvements in physiological indicators. However, its effect on ESE is influenced by participants’ levels of SI, resulting in different outcomes across conditions. For individuals with low SI, ESE improved more under traditional coaching. This may be because traditional instruction emphasizes interpersonal interaction and a sense of accomplishment, which are particularly effective in motivating less-involved participants to engage in training. In contrast, for highly involved individuals, ESE did not change significantly with sports technology assistance. This suggests that their baseline confidence is relatively stable and less affected by external interventions.

In future development, sports technology should focus on addressing the diverse needs of users with different levels of SI through personalized design. For users with low SI, incorporating motivational features such as simplified operation, achievement tracking, and opportunities for social interaction may enhance engagement, build a stronger sense of accomplishment, and improve ESE. For highly involved users, more sophisticated capabilities including precise performance analysis, advanced training monitoring, and real-time feedback would better align with their preference for self-regulation and deeper training engagement.

In addition, integrating AI and ML into sports technology systems would allow them to learn from users’ behavior and performance patterns. This would enable the delivery of dynamic, personalized training programs that improve training efficiency and support long-term development of ESE. Such adaptive systems can enhance long-term adherence and broaden the value of sports technology across different SI levels. Ultimately, these developments may contribute to more inclusive health promotion strategies by encouraging sustainable physical activity participation.

## Supporting information

S1 File Dataset in Excel format. (XLSX)

S2 File Data set in SPSS format. (SAV)
